# Schuurs‐Hoeijmakers syndrome in a patient from Iraq ‐ Kirkuk

**DOI:** 10.1002/ccr3.4897

**Published:** 2021-10-04

**Authors:** Sahar Adnan Abdulqader, Wafaa Abdulqader Wli, Samer Habeeb Qaryaqos

**Affiliations:** ^1^ HD(Psychiatry) MBCHB, Azadi Teaching Hospital Kirkuk Iraq; ^2^ CBAP(Pediatrics) MBCHB, Azadi Teaching Hospital Kirkuk Iraq; ^3^ FIBMS(Radiology) MBCHB, Al‐Hamdanyia General Hospital Mosul Iraq

**Keywords:** coloboma, neonatal seizure, PACS1, Schuurs‐Hoeijmakers syndrome

## Abstract

Schuurs‐Hoeijmakers syndrome is a very rare disorder with less than 60 cases reported worldwide. This is a case report of a patient with SHMS from Iraq, the first in the area of the Middle East. He had epilepsy during his first days of life and a subsequent neurodevelopmental delay.

## Graphical abstract

Schuurs‐Hoeijmakers syndrome is a very rare disorder with less than 60 cases reported worldwide at the time of this report, none in the Middle East area so far! This report will add to the versatile phenotypic and clinical presentations across different populations for better understanding and detecting the disorder.

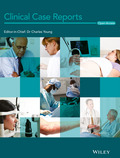

## 1 INTRODUCTION

Schuurs‐Hoeijmakers syndrome (SHMS) also called autosomal dominant intellectual disability 17 is a rare disease characterized by intellectual disability and dysmorphic facial features among various physical abnormalities due to PACS1 mutation.^1^

The PACS1 variant causes an amino acid change from Arg to Trp at position 203.

The syndrome is known to have different manifestations and a range of neurodevelopmental outcomes!

To date, around 60 cases were reported worldwide, mostly in Western populations, few in Asia, but none in the middle east area so far.^2^ ^,^ ^3^ ^,^ ^4^ As common features included a distinctive facial appearance, delayed speech and delayed psychomotor development/ intellectual disability ranging from mild to moderate. Most patients had anomalies in the eyes, nose, heart, and gastrointestinal system.^4^

Here, we present a case of a 3‐year‐old male child who was born with a distinctive facial features and suffered from epilepsy since he was 27 days old! Now at the time of this report, the delay in his psychomotor development along with some autistic features are getting more clear. This may support the hypothesis that was made by Hoshino et al that the PACS1 mutation leads to an inherent dopaminergic insufficiency that underlies the developing symptoms along with the neurodevelopmental processes.^1^

## 2 CASE REPORT

The patient is a 3‐year‐old male child born to non‐consanguineous parents, he is the third and the youngest sibling in the family, and family history is negative regarding neuropsychiatric disorders and congenital malformations.

The pregnancy was uncomplicated, and he was born vaginally at term with a birth weight of (3.200 g) by a certified midwife who reported that he had cyanosis and delayed crying.

The eyes were the first sign noticed by the parents, and clinical examination confirmed that he had bilateral coloboma involving the iris and retina. The pediatrician then advised to do an echocardiography which was normal.

He was admitted to the hospital at age of 5 days because of involuntary movements in the face and upper limbs with hypotonia and was treated as a case of hypoglycemia due to poor feeding. He developed tonic‐clonic seizures when he was 27 days old and was admitted to the hospital again. At the time, he had poor feeding, vomiting, floppiness, and cold limbs.

He was treated as a case of meningitis and received intravenous antibiotics. The seizures were controlled with phenobarbitone 20 mg and sodium valporoate 200 mg syrups along with intravenous benzodiazepines, and he needed maximum for weight dosages to control the seizures.

**FIGURE 1:**
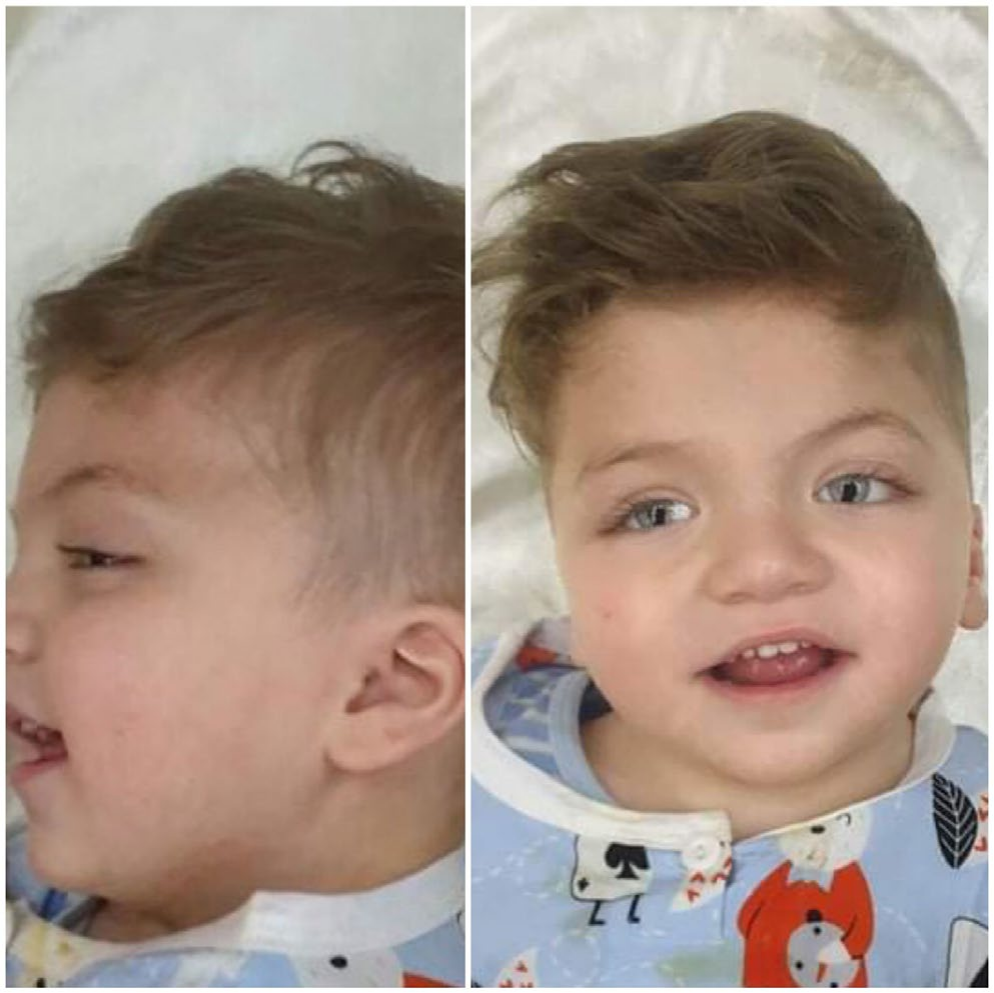
Front and lateral view of the patient at 3 years. he has flat occipit, low set ears, arched eyebrows, long eyelashes, bilateral coloboma, hypertelorism with downslanting palpebral fissures, bulbous nasal tip, wide mouth with downturned corners, smooth philtrum, tented thin upper lip, thin upper vermilion, wide spaced teeth

**FIGURE 2:**
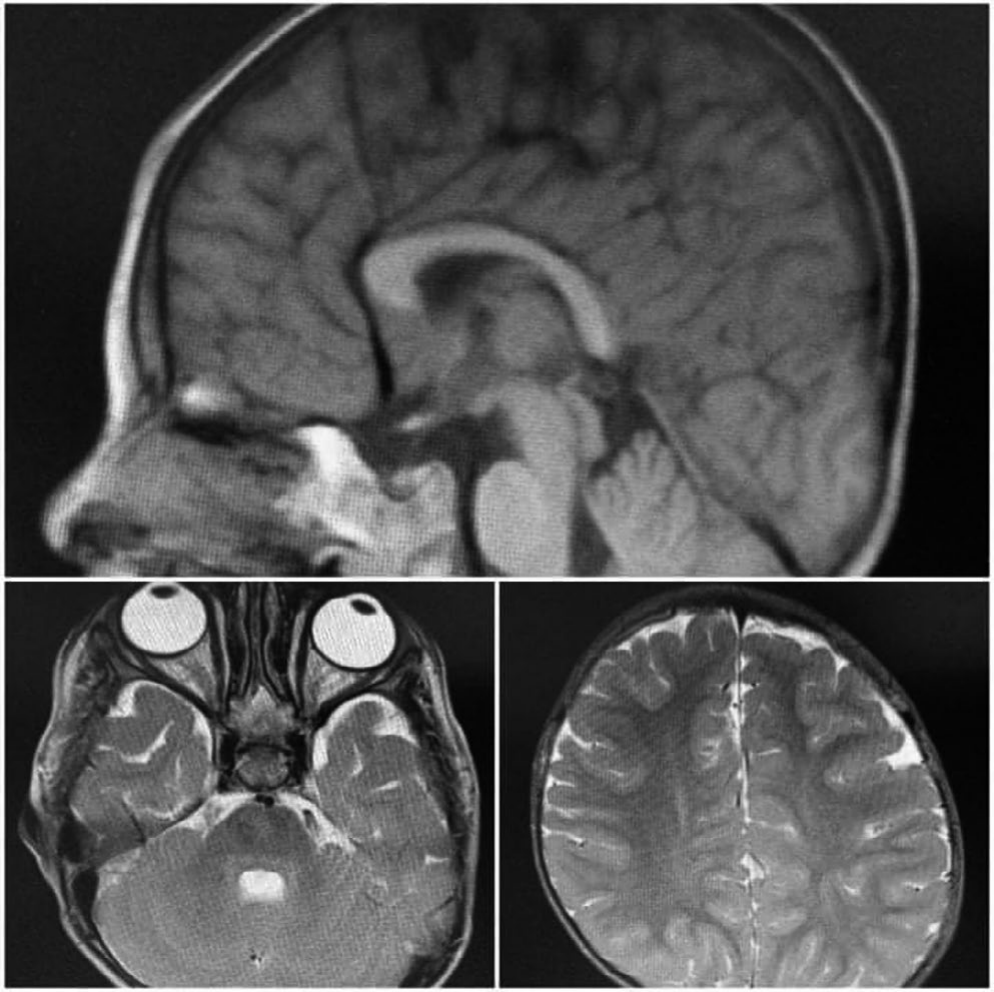
Brain imaging (MRI) shows evidence of mild cerebral atrophy and mild thinning of the corpus callosum with mild bilateral retinal coloboma

He showed dysmorphic facial features: flat occipit, low set ears, arched eyebrows, long eyelashes, bilateral coloboma, hypertelorism with downslanting palpebral fissures, bulbous nasal tip, wide mouth with downturned corners, smooth philtrum, tented thin upper lip, and thin upper vermilion (Figure 1 and 2).

Other bodily features: muscular hypotonia, clinodactyly of the little fingers, the umbilicus was well‐defined hypopigmented plaque, and he had lichenified skin on the sole of both feet. Abdominal and pelvic ultrasonography revealed bilateral cryptorchidism otherwise normal.

Electroencephalogram showed hypsarrthythmic pattern, highly suggestive of generalized epilepsy.

Brain imaging (MRI) revealed evidence of mild cerebral atrophy and mild thinning of the corpus callosum with mild bilateral retinal coloboma.

Cerebrospinal fluid (CSF) analysis was inconclusive.

Then, it was decided from the results of his MRI and the CSF analysis to stop the antibiotics and treat him as a case of brain atrophy with epilepsy. After 1 week of hospital admission, he was discharged on the same antiepileptic syrups.

His parents frequently noticed back arching dystonia, chewing, and twitching movements, uncontrolled laughing, giggling and grimacing when he was 6 months old.

At age of 1 year old, his seizure was under control with levetiracetam syrup 100 mg twice a day, but the family was concerned about his prognosis and the possibility of a metabolic syndrome was raised by many of his physicians. Eventually, they decided to do a genetic study. The whole exome sequence analysis revealed a heterozygous c.607C > T (p. Arg203Trp) mutation in PACS1 gene (NM_018026.3).

When he was 18 months old, he became seizure free, his neurologist stopped his antiepileptic medications, and he was treated mainly for constipation with laxatives. He could only feed on fluids and was easily chocked by food particles at this age.

The bilateral cryptorchidism was surgically corrected by orchidopexy at his 18^th^ month of age.

Postoperative care suggested unsuspected high pain threshold.

At the time of this report, he was 38 months old. He had short stature, wide spaced teeth. He could sit without support and rolled freely across the room with occasional attempts to stand on his feet holding on to furniture.

He made loud noises to draw attention and could only say (baba).

His parents described some behavioral difficulties, as he developed a cling to a certain routine, watching only one specific cartoon and accepting care from his father only who feeds him and holds him most of the time. He showed irritable mood around unfamiliar faces and places expressed mostly as self injurious behavior like head banging and hand biting; otherwise, he was known to possess a friendly disposition.

He showed food aversion to certain types of food especially those with solid particles. The family complained from his habit of swallowing food without chewing it properly which exacerbated his constipation problem.

Some stereotyping behavior was described like purposeless clapping and eyes rolling.

Sometimes the constipation caused him irritability and anger tantrums.

Diet adjustment proved to be helpful.

## 3 DISCUSSION

Schuurs‐Hoeijmakers syndrome (SHMS) is a very rare disorder that is—at least at the time being—impossible to diagnose clinically without a genetic study, which can be expensive for most families. Increasing reports from around the world can bring more attention to this population and spare much time and efforts!

As this rare disorder was not yet described in this part of the world, we hope this case report will be of benefit to better understand the disorder and to confirm its previously described phenotype regardless of ethnicity to make it easier to detect.

## 4 CONCLUSION

This case report sheds light on the difficulties that face the patients and their families and restrain physicians from making a quick and affirmative diagnosis due to lack of facilities in this part of the developing world!

Knowing about the correct diagnosis even with unfavorable prognostic factors can make the path more clear and the journey less mysterious!

## CONFLICT OF INTEREST

The authors have no conflict of interest to declare.

## AUTHOR CONTRIBUTIONS

Sahar Adnan Abdulqader: corresponding author, conceived the idea, led the writing, editing, revising and followed up the patient, Wafaa Abdulqader Wli: contributed to the clinical presentation of the patient and followed his diet adjustment, Samer Habeeb Qaryaqos: contributed to the neuroimaging findings and interpretation.

## ETHICAL APPROVAL

Written informed consent was obtained from the parent to use the data and pictures and publish this report in accordance with journal's patient consent policy.
